# Molecular Evolution of Candidate Genes for Crop-Related Traits in Sunflower (*Helianthus annuus* L.)

**DOI:** 10.1371/journal.pone.0099620

**Published:** 2014-06-10

**Authors:** Jennifer R. Mandel, Edward V. McAssey, Savithri Nambeesan, Elena Garcia-Navarro, John M. Burke

**Affiliations:** 1 Department of Plant Biology, University of Georgia, Athens, Georgia, United States of America; 2 Instituto de Agricultura Sostenible, Consejo Superior de Investigaciones Científicas, Córdoba, Spain; Natural History Museum of Denmark, Denmark

## Abstract

Evolutionary analyses aimed at detecting the molecular signature of selection during crop domestication and/or improvement can be used to identify genes or genomic regions of likely agronomic importance. Here, we describe the DNA sequence-based characterization of a pool of candidate genes for crop-related traits in sunflower. These genes, which were identified based on homology to genes of known effect in other study systems, were initially sequenced from a panel of improved lines. All genes that exhibited a paucity of sequence diversity, consistent with the possible effects of selection during the evolution of cultivated sunflower, were then sequenced from a panel of wild sunflower accessions an outgroup. These data enabled formal tests for the effects of selection in shaping sequence diversity at these loci. When selection was detected, we further sequenced these genes from a panel of primitive landraces, thereby allowing us to investigate the likely timing of selection (i.e., domestication vs. improvement). We ultimately identified seven genes that exhibited the signature of positive selection during either domestication or improvement. Genetic mapping of a subset of these genes revealed co-localization between candidates for genes involved in the determination of flowering time, seed germination, plant growth/development, and branching and QTL that were previously identified for these traits in cultivated × wild sunflower mapping populations.

## Introduction

Strong directional selection is thought to be responsible for the dramatic phenotypic differences between domesticated lineages and their wild progenitors [1], [2]. Genetic map-based approaches, including both QTL and association analyses, have been used to identify numerous genomic regions and, in some cases, genes underlying these phenotypic transitions (e.g., *fw2.2*, [3]; *fw3.2* [4], [5] *tb1*, [6]; *sh4*, [7]). An alternative to mapping studies is the use of molecular population genetic methods to identify genes or genomic regions that may have experienced past selection. These efforts typically involve statistical tests to determine if the observed pattern of genetic diversity in a particular gene or genomic region can be explained by the standard neutral model (e.g., [8]–[13]). Rejection of the null hypothesis of neutrality provides evidence of past selection.

Overall, crop lineages are expected to exhibit a genome-wide loss of genetic diversity relative to their wild progenitors due to the occurrence of population bottlenecks during domestication and/or improvement [2], [14]. But because selection influences genetic diversity in a locus-specific manner, genes targeted by positive selection will exhibit a greater than expected loss of diversity as compared to the genome-wide, neutral expectation [12], [13], [15]. Importantly, this provides a means for identifying genes, or at least genomic regions, that are likely to be of agronomic importance even though they may be recalcitrant to map-based analyses due to a lack of segregating variation within the crop lineage. Here, we describe molecular evolutionary analyses aimed at identifying genes that were targeted by selection during the evolution of cultivated sunflower (*Helianthus annuus* L.) from amongst a pool of candidates identified based on homology to genes of known effect from other study systems.

Cultivated sunflower was domesticated from wild sunflower (both *H. annuus*) approximately 4,000 years ago by Native Americans as a source of edible seeds and as well as for non-food purposes (e.g., as a source of dyes for textiles; [16]). More recently, sunflower has been the subject of intensive breeding as it has been transformed into a globally-important oilseed crop [17]. Wild sunflower exhibits seed dormancy, variable flowering time, extensive and variable branching, and it also produces relatively small seeds that are dispersed upon maturity (i.e., the mature heads “shatter”). In contrast, cultivated sunflower typically exhibits a loss of seed dormancy, more rapid/consistent flowering, strong apical dominance (i.e., a loss of branching), and considerably larger seeds that are retained in the head until harvest. Previous studies have primarily employed map-based approaches to identify genomic regions involved in the evolution of cultivated sunflower (e.g., [18]–[21]), though evolutionary analyses have also shown great promise [22]–[24].

In the present study, we mined the literature for genes from other species that are known to influence traits related to the evolution of cultivated sunflower. These included genes with known effects on floral development and flowering time, seed/fruit development, germination, plant growth/development, and branching. We then identified homologs of these genes in sunflower and sequenced them from panels of wild, primitive, and improved sunflowers. These data allowed us to test for evidence of positive selection during the domestication and/or improvement of sunflower. When possible, we also genetically mapped genes showing evidence of selection and compared their positions to those of QTL that had previously been mapped in cultivated x wild sunflower mapping populations. We found strong evidence for positive selection in a number of these genes as well as evidence of QTL co-localization in several cases. As such, these genes are excellent candidates for future functional studies aimed at understanding the molecular mechanisms underlying the evolution of cultivated sunflower.

## Materials And Methods

### Gene identification and primer design

To identify candidates for genes underlying traits related to sunflower domestication and improvement, we searched the literature for genes influencing relevant aspects of floral development and flowering time, seed/fruit development, seed germination, plant growth/development, and branching. We then performed BLAST searches of these genes against sunflower expressed sequence tags (ESTs) from the Compositae Genome Project EST Database (http://compgenomics.ucdavis.edu/) as well as the reference transcriptome assembly from Bachlava et al. [25]. Reciprocal best BLAST hits with an E-value of less than 10E-10 were identified as putative sunflower homologs and retained for further analysis (Table S1). Primers specific to a portion of each sunflower unigene identified via BLAST were then designed using either PrimerPlus 3.0 (http://www.bioinformatics.nl/) or PrimerQuest (http://www.idtdna.com/). To help avoid designing primers across splice sites, we used a tblastx-based intron finding Perl script (http://www.citrusgenome.ucr.edu/usa/ucr/Files.php) with *Arabidopsis* genome sequence information (v. 10) available from TAIR (http://www.arabidopsis.org/) to identify putative intron positions in sunflower unigenes. The resulting primer sequences can be found in Table S2. We also included a set of 11 presumptively neutral control genes that were previously identified by Chapman et al. [22] (Table S2).

### Plant materials and DNA sequencing

The focus of this study was a collection of 28 *H. annuus* individuals including 8 wild sunflowers, 6 primitive domesticates (i.e., Native American landraces which represent an intermediate stage between wild sunflower and modern cultivars), and 14 improved lines (Table 1). Achenes for these individuals were obtained, with permissions, from the USDA North Central Regional Plant Introduction Station (NCRPIS) and French National Institute for Agricultural Research (INRA). The improved lines included the parents of a well-characterized sunflower recombinant inbred line (RIL) mapping population (RHA 280 and RHA 801) as well as the “Core 12” from Mandel et al. [26], which includes 12 inbred lines that capture ca. 50% of the allelic diversity present within the cultivated sunflower gene pool. We also included two outgroups: *H. argophyllus*, which is sister to *H. annuus*, and *H. petiolaris*, another closely related species that is sister to the *H. annuus*/*H. argophyllus* clade. A single individual from each of the 28 genotypes was grown to the seedling stage, and total DNA was extracted from each using a CTAB extraction protocol [27] and quantified using Picogreen (Applied Biosystems). The quantity/quality of DNA was also evaluated using a Nanodrop 1000 spectrophotometer. For candidate gene sequencing, we employed a tiered approach, as follows: (1) we first sequenced all candidate genes in the improved panel; (2) following the recommendations of Yamasaki et al. [28] and Chapman et al. [29], those candidates that had no or very low nucleotide diversity (*π*<0.01) in the improved panel were retained for sequencing in the wild panel and the outgroups, thereby enabling tests for selection; and (3) when a gene showed evidence for selection in the improved panel, it was also sequenced in the panel of primitive accessions. This last piece of data allowed us to further infer the likely timing of selection (i.e., domestication vs. improvement by investigating these genes in a panel containing primitive sunflower varieties vs. recently improved sunflower varieties).

**Table 1:** List of sunflower accessions analyzed in this study.

Panel	Name	USDA Accession Number
*Wild*	Ames 14400	PI 649851
	Ann-1114	PI 613727
	Ames 1473	PI 413027
	Ames 1455	PI 413011
	Ames 1516	PI 413067
	Ames 23238	PI 649853
	Ames 23940	PI 649854
	Ann-646	PI 435552
*Primitive*	Havasupai	PI 369358
	Hidatsa	PI 600721
	Hopi	PI 432504
	Mandan	PI 600717
	Maiz Negro	PI 650761
	Seneca	PI 369360
*Improved*	Mammoth	PI 476853
	HA 234	PI 599778
	HA 316	NSL 208764
	HA 404	PI 597368
	HA 821	PI 599984
	RHA 280	PI 552943
	RHA 328	NSL 202284
	RHA 358	PI 531071
	VIR 847	PI 386230
	RHA 408	PI 603989
	RHA 426	PI 617099
	RHA 801	PI 599768
	SF 33*	---
	SF 230*	---

*Accessions from the French National Institute for Agricultural Research.

PCR was performed in a total volume of 20 µL containing 5 ng of template DNA, 30 mM Tricine pH 8.4-KOH, 50 mM KCl, 2 mM MgCl_2_, 125 µM of each dNTP, 0.2 µM reverse primer, 0.2 µM forward primer and 2 units of *Taq* polymerase. The PCR conditions involved a ‘touchdown’ protocol, as follows: 3 min at 95C; 10 cycles of 30 s at 94C, 30 s at 65C and 45 s at 72C, annealing temperature decreasing to 55C by 1C per cycle, followed by 30 cycles of 30 s at 94C, 30 s at 55C, 45 s at 72C, followed by 20 min at 72C. PCR products were checked for single-banded amplification via electrophoresis on 1% agarose gels. Amplification conditions were modified for loci that exhibited weak or non-specific amplification (i.e., faint or multiple bands, respectively) by either decreasing (i.e., starting at 60C and descending to 50C) or increasing (i.e., starting at 70C and descending to 60C) the annealing temperature.

PCR products were treated with 4 units Exonuclease I and 0.8 units Shrimp Alkaline Phosphatase (USB) at 37C for 45 min followed by enzyme denaturation at 80C for 15 min to prepare for sequencing using BigDye v3.1 (Applied Biosystems). The sequencing reactions were cleaned using Sephadex (Amersham) before being run on an ABI 3730xl (Applied Biosystems). In cases where direct sequencing results were unclear due to unresolvable heterozygous bases, indels, or short repeats, PCR products were TA-cloned into pGEM-T vectors (Promega), transformed into competent *Escherichia coli* (JM109; Promega), and screened for the presence of an insert. At least five positive colonies per individual were then sequenced as above except that vector primers [T7 and SP6] were used. Sequences have been deposited in the National Center for Biotechnology Information (NCBI) as BioProject PRJNA248055.

### Sequence analyses and tests for selection

For all genes, sequences were aligned using Sequencher version 4.10 (GeneCodes), and FASTA files were generated for each. These FASTA files were then imported into DnaSP version 4.50.2 [30] for analysis. Where possible, individuals exhibiting heterozygous bases were resolved into haplotypes using the PHASE algorithm in DnaSP (or they were cloned and re-sequenced; see above). We then used DnaSP to compute the number of synonymous segregating sites (S), synonymous nucleotide diversity (*π*), Watterson's [31] estimate of synonymous diversity (*θ*), number of segregating indels, and the synonymous genetic distance from the outgroup for each gene. The distance (D), from the outgroup was determined by calculating the number of synonymous segregating sites in all pairwise comparisons among sequenced individuals within each panel and the outgroup and then averaging to obtain D (the authors of the program recommend using one sequence from each species; we used all individuals within a panel in order to provide a more robust value for D). We also compared levels of genetic diversity amongst the three panels: wild, primitive (for those genes with preliminary evidence of selection), and improved. For these comparisons, S and the angular transformation of *π* and *θ* values were analyzed. For all genes that were sequenced in all three panels, two-factor ANOVAs (panel and locus) were performed using JMP version 9 (SAS Institute, Cary, NC) and posterior Tukey-Kramer tests were used to test for significant differences amongst means.

For the selection analyses, we used the maximum likelihood (ML) version [32] of the Hudson-Kreitman-Aguade (HKA) test [33], which allows for an explicit test of selection at individual loci in a multilocus framework. The neutral theory of molecular evolution [34] predicts that the amount of within-species polymorphism should be correlated with levels of between-species divergence. The ML-HKA test evaluates this prediction in a locus-specific fashion, thereby allowing for the identification of individual genes showing evidence of selection. It does this by comparing the fit of two models to the observed data. In one model, all loci (a set of neutral controls +a locus of interest) are assumed to be evolving neutrally. In the second model, the locus of interest is deemed to be under selection. Significance is then evaluated by comparing twice the difference in the likelihoods of the two models against a chi-square distribution with one degree of freedom [35].

We first confirmed that the collection of neutral genes utilized herein were not themselves under selection. To do this, we performed a “round-robin” test of all 11 putative neutral genes against each other to confirm selective neutrality (i.e., that selection has not influenced their pattern of nucleotide polymorphism). For each gene, this entailed using the ML-HKA test to compare two models, one model in which all 11 genes were assumed to be neutral, and one in which 10 genes are neutral and one is selected. This process was repeated with five different seed numbers for each of the 11 putatively neutral genes in each of the panels and none exhibited evidence of selection.

Following confirmation that our control loci were indeed behaving in a neutral fashion, we performed the ML-HKA test for each of the candidate genes of interest. This initially involved comparing the levels of polymorphism and divergence in the wild panel and the improved panel. As noted above, when a gene showed significant evidence of positive selection in the improved lines, we also sequenced it in the primitive panel and tested for selection at that stage. All ML-HKA tests were performed with five different seed numbers and a chain length of 100,000 as recommended by the authors. The resulting maximum likelihoods were averaged across the five replicates and used to perform a likelihood-ratio test.

### Genetic mapping of candidate genes

Candidate genes that demonstrated evidence of positive selection during domestication and/or improvement were screened for polymorphism in eight arbitrarily chosen individuals of one of two recombinant inbred line (RIL) mapping populations via Sanger sequencing: an improved x wild sunflower cross (HA89 x ANN1238; [18], [36]) for domestication candidates and an improved x primitive sunflower cross (NMS373 x Hopi; [37]) for improvement candidates. When a mappable polymorphism was identified, the locus was amplified from a larger set of 96 RILs from the appropriate cross and scored as either a length variant or via PCR-restriction fragment length polymorphism (PCR-RFLP). Loci were added to previously published linkage maps using either the data and methods of Baack et al. [36] for the HA89 x ANN1238 RILs or Bowers et al. [37] for the NMS373 x Hopi RILs. If no polymorphism in the amplified region was readily identified, we used BLAST similarity of the selected candidate genes to the consensus genetic map of Bowers et al. [37] or to an existing sequence-based map [38] to locate the genomic position of the locus. When possible, we also compared the genetic map positions of genes under selection to those of previously mapped QTL for the phenotypes of interest. This was done by projecting QTL from several previous studies [18], [19], [39], [40] onto the sunflower consensus map [37] based on shared markers. For the QTL mapping populations of Burke et al. [18] and Wills et al. [19], we used the sunflower CMap database (http://sunflower.uga.edu/cmap/) to identify markers that flanked the QTL of interest (i.e, those located near candidate genes for the same trait) and were also present on the sunflower consensus map [37]. We then used the genetic positions from the consensus map to display the QTL relative to the positions of the candidate genes (see below; note: when available, we used the 2-LOD interval for marking QTL regions as presented in the studies). The studies of Dechaine et al. [39] and Brunick [40] were not included in the CMap database, so the shared flanking markers were identified from the original source documents and projected as above.

## Results And Discussion

**Figure 1:**
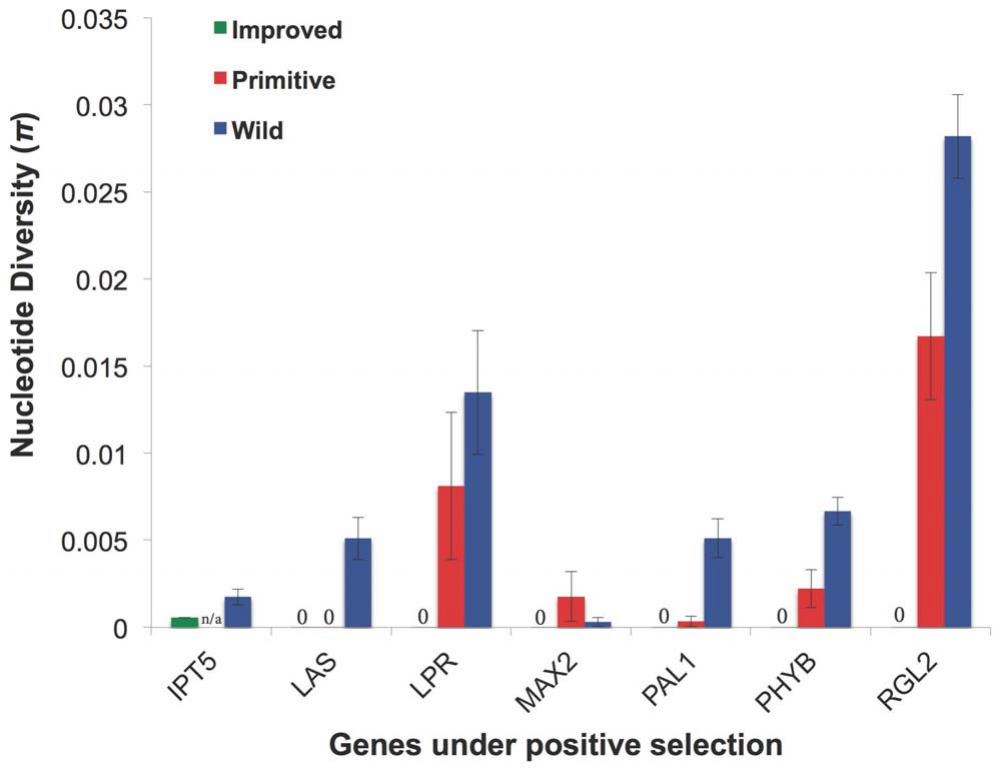
Nucleotide diversity in wild, primitive, and improved sunflower for the genes shown to be under positive selection during the evolution of cultivated sunflower. Error bars indicate standard deviations. Note that we were unable to obtain sequence information for the *IPT5* gene in the primitive panel.

As expected, we observed an overall, progressive loss of genetic diversity in the primitive and improved sunflower lines as compared to their wild progenitor (Table 2). Of the 76 candidate genes that we sequenced, 24 exhibited little or no nucleotide variation in a panel of 14 improved sunflower lines (π<0.0202, though most of these 24 were much lower than this value; see Table 3 and Table S3 for additional statistics), consistent with the possibility that they experienced positive selection during domestication and/or improvement. We then sequenced these 24 genes in a geographically diverse panel of eight wild sunflower individuals as well as outgroups and used the resulting data to test for selection. Seven of these genes exhibited significant departures from neutrality in the ML-HKA tests (Table 3; Figure 1), thereby providing strong evidence of past selection. Note also that some of the candidate genes harbored low genetic diversity in the wild panel as compared to the neutral genes. In fact, two candidate genes for branching (*IPT5* and *MAX2*) were shown to be under selection in the wild. After sequencing these genes in the primitive landraces, we found that *LATERAL SUPPRESSOR* (*LAS*) showed evidence for selection in both the primitive and improved lines. Five other genes, including *LOW PHOSPHATE ROOT* (*LPR*), *MORE AXILLARY GROWTH 2* (*MAX2*), *PHENYLALANINE AMMONIA-LYASE 1* (*PAL1*), *PHYTOCHROME B (PHYB*), and *RGA-LIKE* 2 (*RGL2*), showed evidence of selection in the improved lines only. We thus conclude that *LAS* likely experienced selection during the initial phase of sunflower domestication, whereas selection on the remainder was likely restricted to the subsequent period of improvement. Note that a reliable PCR product was not obtained for *ISOPENTENYLTRANSFERASE 5* in the primitive lines. We were thus unable to investigate the timing of selection for this gene.

**Table 2:** Population genetic diversity statistics for candidate and neutral/control genes.

Type	Panel	N	S (SE)	Sig.^1^	π (SE)	Sig.^1^	θ (SE)	Sig.^1^
***Candidate***	W	24	6.25 (1.48)	X	0.022 (0.005)	X	0.024 (0.005)	X
	P	6	3.77 (1.68)	XY	0.012 (0.008)	XY	0.013 (0.006)	XY
	I	24	0.91 (0.33)	Y	0.0028 (0.001)	Y	0.0029 (0.001)	Y
***Neutral***	W	11	7.18 (1.55)	A	0.039 (0.006)	A	0.043 (0.008)	A
	P	11	3.55 (0.93)	B	0.027 (0.005)	B	0.025 (0.005)	B
	I	11	3.27 (1.10)	B	0.020 (0.005)	B	0.014 (0.004)	C

Panel: W =  wild, P =  primitive, I =  improved; N =  number of genes sequenced; S =  number of segregating synonymous sites; π =  nucleotide diversity for synonymous sites; θ =  Waterson's theta for synonymous sites; Sig.  =  results of posterior Tukey-Kramer test with different letters representing significantly different values. ANOVA results: Neutral genes: *θ* (*F*_2, 20_ = 8.58, *p*<0.0001), π (*F*_2, 20_ = 8.73, *p*<0.0001), S (*F*_2, 20_ = 4.89, *p* = 0.0009). Candidate genes: π (*F*_2,__10_ = 5.26, *p* = 0.001), *θ* (*F*_2,__10_ = 5.19, *p* = 0.01), S, (*F*_2,__10_ = 3.56, *p* = 0.03).

1 ANOVA for candidate genes performed on the six candidate genes that were sequenced in all three panels.

**Table 3:** Population genetic diversity statistics for the invariant genes tested for evidence of positive selection.

Gene	Trait	Panel	L	*l*	S	π	θ	Sig.
*ACTIN-RELATED PROTEIN 7*	Pleiotropic effects on	W	549	48.67	2	0.01761	0.01585	ns
	plant development	P	-	-	-	-	-	-
		I	549	48.67	0	0	0	ns
*BLIND*	Branching	W	383	12.17	0	0	0	ns
		P	-	-	-	-	-	-
		I	383	12.17	0	0	0	ns
*CHALCONE SYNTHASE*	Plant growth; regulation	W	310	40.81	0	0	0	ns
	of auxin	P	-	-	-	-	-	-
		I	310	40.67	0	0	0	ns
*CONSTANS*	Flowering	W	483	114.17	9	0.03213	0.02787	ns
		P	-	-	-	-	-	-
		I	483	114.11	4	0.00518	0.00919	ns
*CYTOKININ OXIDASE/*	Plant growth	W	375	88.67	3	0.01015	0.0136	ns
*DEHYDROGENASE 1*		P	-	-	-	-	-	-
		I	375	88.67	0	0	0	ns
*ELONGATION FACTOR*	Plant growth	W	380	37.98	2	0.00943	0.01587	ns
*BINDING SITE 1B*		P	-	-	-	-	-	-
		I	380	38	0	0	0	ns
*FASCIATED*	Fruit development	W	435	43.17	0	0.02978	0.0268	ns
		P	-	-	-	-	-	-
		I	435	43.17	0	0	0	ns
*GLABRA 2*	Seed development	W	639	88.31	7	0.028370.02493	0.02493	ns
		P	-	-	-	-	-	-
		I	639	89	4	0.0124	0.01204	ns
*GLABRA2-EXPRESSION*	Cell fate/division affecting	W	625	90.04	0	0	0	ns
*MODULATOR*	plant development	P	-	-	-	-	-	-
		I	625	90.58	0	0	0	ns
*HEADING DATE 6*	Flowering	W	737	38.67	0	0	0	ns
		P	-	-	-	-	-	-
		I	737	38.67	0	0	0	ns
*INOSITOL POLYPHOSPHATE*	Branching	W	367	86.11	11	0.02669	0.0385	ns
*6-/3-KINASE 2B*		P	-	-	-	-	-	-
		I	367	85.98	2	0.00391	0.00598	ns
***ISOPENTENYLTRANSFERASE 5***	**Plant growth**	**W**	**486**	**116.52**	**1**	**0.00107**	**0.00108**	*
		**P**	**N/A**	**N/A**	**N/A**	**N/A**	**N/A**	**N/A**
		**I**	**486**	**116.50**	**0**	**0**	**0**	**
***LATERAL SUPPRESSOR***	**Branching**	**W**	**532**	**103.67**	**7**	**0.02244**	**0.02035**	**ns**
		**P**	**532**	**103.67**	**0**	**0**	**0**	*
		**I**	**532**	**103.67**	**0**	**0**	**0**	**
***LOW PHOSPHATE ROOT***	**Root growth/development**	**W**	**413**	**97.75**	**10**	**0.02774**	**0.03391**	**ns**
		**P**	**413**	**98.06**	**11**	**0.01868**	**0.03715**	**ns**
		**I**	**413**	**98**	**0**	**0**	**0**	***
***MORE AXILLARY GROWTH 2***	**Branching**	**W**	**468**	**86.5**	**1**	**0.00165**	**0.00364**	*
		**P**	**468**	**86.44**	**4**	**0.00774**	**0.01532**	**ns**
		**I**	**468**	**86.5**	**0**	**0**	**0**	**
*MORE AXILLARY GROWTH 4*	Branching	W	104	0	-	-	-	-
		P	-	-	-	-	-	-
		I	104	0	-	-	-	-
*METHYL-CPG BINDING 9*	Branching	W	227	52.25	11	0.05391	0.06345	ns
		P	-	-	-	-	-	-
		I	225	52.37	4	0.0202	0.0202	ns
***PHENYLALANINE***	**Plant growth**	**W**	**473**	**105.54**	**12**	**0.02178**	**0.03427**	**ns**
***AMMONIA-LYASE 1***		**P**	**473**	**105.5**	**1**	**0.00158**	**0.00314**	**ns**
		**I**	**473**	**105.5**	**0**	**0**	**0**	*
*PHYTOCHROME A*	Flowering	W	447	46.5	2	0.02085	0.01424	ns
		P	-	-	-	-	-	-
		I	447	46.5	0	0	0	ns
***PHYTOCHROME B***	**Flowering**	**W**	**683**	**166.83**	**13**	**0.02648**	**0.02639**	**ns**
		**P**	**683**	**166.83**	**5**	**0.00908**	**0.00992**	**ns**
		**I**	**683**	**166.83**	**0**	**0**	**0**	**
*PHYTOCHROME E*	Flowering	W	457	103.48	14	0.0379	0.04254	ns
		P	-	-	-	-	-	-
		I	457	103.42	5	0.01029	0.01267	ns
*PIN-FORMED 1*	Root/shoot development	W	411	101.92	11	0.02743	0.03253	ns
		P	-	-	-	-	-	-
		I	411	101.93	2	0.0063	0.00504	ns
*PHOTOPERIOD -H1*	Flowering	W	246	50.31	3	0.017560.01797	0.01797	ns
		P	-	-	-	-	-	-
		I	246	50.33	1	0.00841	0.00511	ns
***RGA-LIKE 2***	**Germination**	**W**	**377**	**89.5**	**31**	**0.11417**	**0.12297**	**ns**
		**P**	**377**	**89.06**	**13**	**0.07069**	**0.04834**	**ns**
		**I**	**377**	**88.83**	**0**	**0**	**0**	***

Panel, W =  wild, P =  primitive, I =  improved; L =  alignment length in basepairs; l =  number of synonymous sites; S =  number of segregating synonymous sites; π =  nucleotide diversity for synonymous sites; θ =  Waterson's theta for synonymous sites; Sig.  =  ML-HKA significance: ns  =  not significant, P<0.001 =  ***, P<0.01 =  **, P<0.05 = *. Bold genes are those that showed significant evidence of selection. Note: we were unable to successfully sequence the *IPT5* gene in P.

One of the selected genes, *PHYB*, is a photoperiod response gene that is thought to play a role in the transition from vegetative to reproductive growth (e.g., [41], [42]). This gene also has a possible role in seed germination [43] (see discussion of germination and seed dormancy in sunflower below). The control of flowering is an important agricultural trait, and the evolution of flowering time is known to have played a critical role in the success of many crop species, including sunflower [17], [23], [44], [45]. Wild sunflower exhibits extensive variation in flowering time [46], [47], whereas the primitive sunflowers typically flower later in the season [48], and modern varieties have been selected for relatively early flowering [44], [49] making it possible to produce sunflower across a broader range of environments [17].

**Figure 2:**
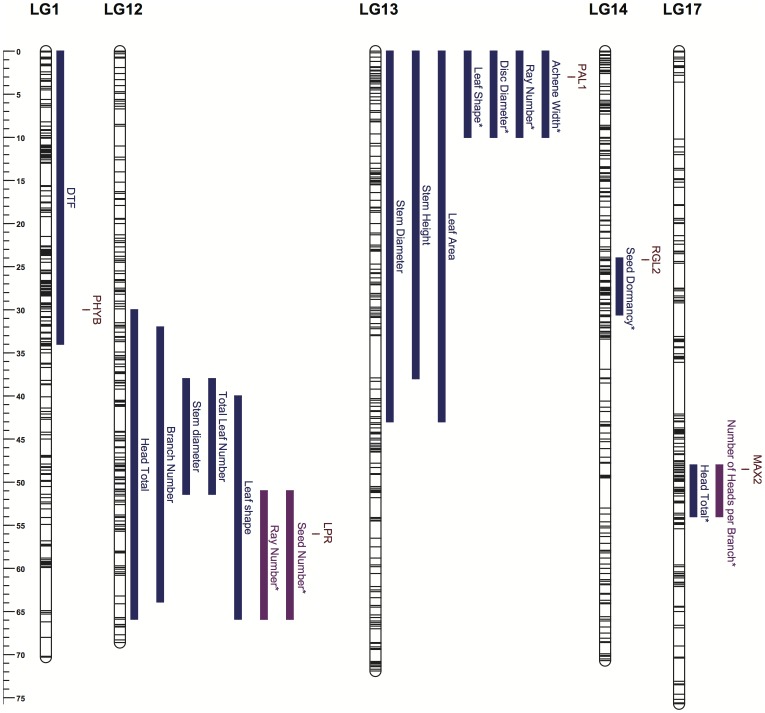
Co-localization of candidates for genes under selection and QTL for flowering time, branching, germination. , and a number of plant growth and development traits that were previously identified in an improved x wild (shown in blue) or in both an improved x wild and a primitive x wild (shown in purple) sunflower QTL mapping population. QTL presented as 1-LOD are marker with an asterisk. All QTL and candidate gene positions are presented in the context of the sunflower consensus map (Bowers et al. 2012).

Interestingly, Blackman et al. [23] included *PHYB* in their analysis of the role of flowering time genes in the evolution of cultivated sunflower and found marginally significant evidence for selection during improvement (*P* = 0.07). Though *PHYB* was found to have identical predicted protein sequences in the parents of an improved x wild sunflower mapping population that exhibits extensive variation in flowering time, this gene was consistently expressed at higher levels in the cultivar parent. We also found that *PHYB* co-localized with a previously identified QTL in an improved x wild sunflower mapping study ([39]; Figure 2). These results suggest that post-domestication selection may have targeted a *cis*-regulatory element that influences *PHYB* expression and that the diversity loss within *PHYB* itself is a byproduct of this selection.

Like flowering time, plant architecture changed dramatically during the evolution of cultivated sunflower. Initially, selection for increased apical dominance is thought to have resulted in a complete loss of branching [48], [50], [51]. In the mid-20^th^ century, however, apical branching was re-introduced in a subset of cultivated sunflower lines as part of a transition to hybrid breeding and a concomitant desire to produce male lines with indeterminate flowering [52]. Consequently, branching is polymorphic within the cultivated sunflower gene pool. This re-introduction of branching is, however, due to the effects of a recessive allele at a single locus that maps to the upper third of linkage group (LG) 10 [53]. As such, other branching-related genes that were targeted by selection during sunflower domestication or improvement would still be expected to harbor low diversity. In fact, homologs of three genes known to influence branching in other species exhibited evidence of positive selection in sunflower, including one during domestication (*LAS*), one during improvement (*MAX2*), and one with unknown timing (*IPT5*) (Figure 1). *LAS* is a transcription factor and a positive regulator of bud, or branch, initiation [54]–[56], *MAX2* is an F-box protein that is thought to influence lateral shoot growth [57], and *IPT5* is known to be involved in cytokinin biosynthesis [58]. The genomic locations of *LAS* and *IPT5* could not be determined in this study, but *MAX2* (which also showed evidence of selection in wild sunflower) co-localized with a previously identified QTL for branching on LG 17.

Of the genes selected for analysis due to their potential role in other aspects of plant growth and development, two (*LPR* and *PAL1*) showed evidence of positive selection during improvement. *LPR* is a multicopper oxidase affecting root growth/development in *Arabidopsis* [59], whereas *PAL1* is a component of the phenylpropanoid pathway having broad effects on plant growth/development [60]. These genes co-localized with previously known QTL for numerous plant growth traits in sunflower including inflorescence size, plant architecture, leaf shape, and seed size [18], [19], [36] (Figure 2), though a better understanding of the likely phenotypic effects of variation at these genes awaits further study. Finally, *RGL2*, which is a DELLA protein that represses germination in *Arabidopsis* [61], exhibited evidence of selection during improvement. Wild sunflower exhibits strong seed dormancy whereas the primitive and improved varieties have little or no dormancy [40]. Interestingly, *RGL2* co-localized with a QTL for seed dormancy in an improved x wild sunflower cross [40] (Figure 2). These findings make this genomic region, and the *RGL2* gene in particular, a promising target for functional studies involving seed dormancy and germination.

Of course, it is always possible that genes bearing the signature of selection such as those identified above were not themselves targeted by selection. Rather, these genes may simply be linked to the actual targets of selection (i.e., genetic hitchhiking). Though early studies of linkage disequilibrium (LD) in sunflower found that it decayed relatively rapidly (e.g., [62], [63]), more recent analyses have revealed the presence of extended islands of LD within the genome [21]. Importantly, none of our mapped genes that exhibit evidence of selection fall within regions that exhibit elevated LD in the sunflower genome. This pattern of selected genes falling in genomic regions with lower overall LD, along with multiple instances of co-localization with QTL for crop-related traits supports the notion that they were indeed targeted by selection. Through the joint application of molecular population genetic analyses and trait-based mapping approaches, we have thus identified a set of promising loci for future functional studies aimed at understanding the molecular basis of sunflower evolution.

## Acknowledgments

We would like to thank Eleni Bachlava and Jeff Roeder for assistance in the laboratory and Mark Chapman for help with the selection analyses. We would also like to thank two anonymous reviewers for comments on a previous version of this manuscript.

## Supporting Information

Table S1 **List of candidate genes, reference taxa where the gene was identified, and functional traits of interest.**
(DOCX)

Table S2 **Primer sequences and information for candidate genes and control loci (second tab).**
(XLSX)

Table S3 **Statistics for candidate and neutral genes for wild, primitive, and improved individuals.**
(XLSX)
