# Supplementary material for: Molecular Evolution of Candidate Genes for Crop-Related Traits in Sunflower (Helianthus annuus L.)
Source: PLoS One. 2014 Jun 10;9(6):e99620. doi: 10.1371/journal.pone.0099620 (PMC4051887; doi:10.1371/journal.pone.0099620)
Supplement: Table S1 — List of candidate genes, reference taxa where the gene was identified, and functional traits of interest. (DOCX) [file pone.0099620.s001.docx]

Supplementary Table 1. List of candidate genes, reference taxa where the gene was identified, and functional traits of interest.

| Gene Name | Reference Species | Function/Trait | Citation |
| --- | --- | --- | --- |
| *4-COUMARATE:COA LIGASE 1* | *Arabidopsis* | Cell wall growth; lignin deposition | [1] |
| *ACTIN-RELATED PROTEIN 7* | *Arabidopsis* | Pleiotropic effects on plant development | [2] |
| *AGAMOUS-LIKE 20* | *Arabidopsis* | Flowering | [3] |
| *APETALA 2* | *Arabidopsis* | Flower/seed development | [4] |
| *ARABIDOPSIS THALIANA CENTRORADIALIS* | *Arabidopsis* | Flowering | [5] |
| *AUXIN RESISTANT 2* | *Arabidopsis* | Pleiotropic effects on plant growth | [6] |
| *AUXIN RESISTANT 5* | *Arabidopsis* | Plant development/growth | [7] |
| *AUXIN-RESISTANCE 1* | *Arabidopsis* | Branching | [8] |
| *B-BOX DOMAIN PROTEIN 24* | *Arabidopsis* | Photomorphogenesis | [9] |
| *BEL 1-LIKE HOMEODOMAN 2* | *Arabidopsis* | Seed development | [10] |
| *BEL 1-LIKE HOMEODOMAN 4* | *Arabidopsis* | Seed development | [10] |
| *BLIND* | Tomato | Branching | [11] |
| *BRANCHED 1* | *Arabidopsis* | Branching | [12] |
| *BRASSICA RAPA MEMBRANE OCCUPATION AND RECOGNITION NEXUS* | *Brassica rapa* | Plant growth rate | [13] |
| *BRIX9-2-5* | Tomato | Fruit development | [14] |
| *BUSHY AND DWARF1* | *Arabidopsis* | Branching | [15] |
| *CASEIN KINASE 2 α* | *Arabidopsis* | Flowering | [16] |
| *CHALCONE SYNTHASE* | *Arabidopsis* | Plant growth; regulation of auxin transport | [1] |
| *COLORLESS NON-RIPENING* | Tomato | Fruit development | [17] |
| *CONSTANS* | *Arabidopsis* | Flowering | [18] |
| *CYCLOIDEA* | *Antirrhinum majus* | Floral development/shape | [19] |
| *CYTOKININ OXIDASE/DEHYDROGENASE 1* | Rice | Plant growth | [20] |
| *CYTOKININ OXIDASE DEHYDROGENASE 2* | Rice | Plant growth | [20] |
| *EARLY HEADING DATE 1* | Rice | Flowering | [21] |
| *ELONGATION FACTOR BINDING SITE 1B* | *Arabidopsis* | Plant growth | [22] |
| *EMBRYONIC FLOWER 2* | *Arabidopsis* | Flowering | [23] |
| *FASCIATED* | Tomato | Fruit development | [24] |
| *FLAVIN-BINDING, KELCH REPEAT, F-BOX* | *Arabidopsis* | Flowering | [25] |
| *FRUCTOSIDASE 4* | Multiple | Plant growth | [26] |
| *FRUIT WEIGHT 2.2* | Tomato | Fruit development | [27] |
| *GALACTURONOSYL TRANSFERASE 12* | *Arabidopsis* | Plant growth/development | [28] |
| *GIGANTEA* | *Arabidopsis* | Flowering | [29] |
| *GLABRA 2* | *Arabidopsis* | Seed development | [30] |
| *GLABRA 2-EXPRESSION MODULATOR* | *Arabidopsis* | Cell fate/division affecting  plant development | [31] |
| *GLUTAMYL-TRNA REDUCTACE* | *Arabidopsis* | Plastid development;  chloroplast biogenesis | [32] |
| *HEADING DATE 6* | Rice | Flowering | [33] |
| *HEAVY METAL ATPASE 5* | *Arabidopsis* | Plant growth | [34] |
| *INOSITOL POLYPHOSPHATE 6-/3-KINASE 2B* | *Arabidopsis* | Branching | [35] |
| *ISOPENTENYLTRANSFERASE 4* | *Arabidopsis* | Root formation;  cell division in developing seeds | [36] |
| *ISOPENTENYLTRANSFERASE 5* | *Arabidopsis* | Plant growth | [36] |
| *ISOPROPYL MALATE ISOMERASE LARGE SUBUNIT 1* | *Arabidopsis* | Plant growth | [37] |
| *KIP-RELATED PROTEIN 7* | *Arabidopsis* | Plant growth | [38] |
| *KNOTTED-LIKE HOMEOBOX OF ARABIDOPSIS THALIANA 7* | *Arabidopsis* | Plant growth /development | [39] |
| *LATERAL SUPPRESSOR* | *Arabidopsis* | Branching | [40] |
| *LOW PHOSPHATE ROOT* | *Arabidopsis* | Root growth/development | [41] |
| *METHYL-CPG BINDING 9* | *Arabidopsis* | Branching | [42] |
| *MONOPTEROS* | *Arabidopsis* | Pleiotropic developmental effects | [43] |
| *MORE AXILLARY GROWTH 1* | *Arabidopsis* | Branching | [44] |
| *MORE AXILLARY GROWTH 2* | *Arabidopsis* | Branching | [44] |
| *MORE AXILLARY GROWTH 3* | *Arabidopsis* | Branching | [45] |
| *MORE AXILLARY GROWTH 4* | *Arabidopsis* | Branching | [46] |
| *NO APICAL MERISTEM* | Rice | Branching | [47] |
| *PHENYLALANINE AMMONIA-LYASE1* | *Arabidopsis* | Plant growth | [48] |
| *PHOTOPERIOD -H1* | Barley | Flowering | [49] |
| *PHOTOPERIOD-INDEPENDENT EARLY FLOWERING 1* | *Arabidopsis* | Flowering | [50] |
| *PHYTOCHROME A* | *Arabidopsis* | Flowering, germination | [51,52] |
| *PHYTOCHROME-AND FLOWERING TIME 1* | *Arabidopsis* | Flowering | [53] |
| *PHYTOCHROME B* | *Arabidopsis* | Flowering | [52,54] |
| *PHYTOCHROME C* | *Arabidopsis* | Flowering | [55] |
| *PHYTOCHROME E* | *Arabidopsis* | Flowering | [56] |
| *PIN-FORMED 1* | *Arabidopsis* | Root/shoot development | [57] |
| *REDUCED HEIGHT B1* | Bread Wheat | Plant growth | [58] |
| *REPRESSOR OF GIBBERELLIC ACID 1-3* | *Arabidopsis* | Flowering | [59] |
| *REVOLUTA* | *Arabidopsis* | Branching | [60] |
| *RGA-LIKE 2* | *Arabidopsis* | Germination | [61] |
| *SET DOMAIN GROUP 8* | *Arabidopsis* | Branching | [62] |
| *SHATTERING 1* | Rice | Seed shattering | [63] |
| *SHOOT GRAVITROPISM 7* | *Arabidopsis* | Root/shoot development | [64] |
| *SHORT ROOT TRANSCRIPTION FACTOR* | *Arabidopsis* | Root growth/development | [65] |
| *SQUAMOSA PROMOTOER BINDING-LIKE 12* | *Arabidopsis* | Flowering | [66] |
| *SUCROSE-PROTON SYMPORTER 2* | *Arabidopsis* | Seed development; plant growth | [67] |
| *SUPERSHOOT* | *Arabidopsis* | Branching | [68] |
| *TB1, CYC, PCF- GENE* FAMILY 8* | *Arabidopsis* | Leaf development | [69] |
| *VACUOLAR INVERTASE1* | *Arabidopsis* | Root growth | [70] |
| *VERNALIZATION INSENSITIVE 3-LIKE* | *Arabidopsis* | Flowering | [71] |
| *WAXY* | Maize | Fruit development | [72] |

Note that while many of these genes are known to be involved in a variety of functions and have been the subject of numerous studies, we list only the function/trait and corresponding citation that prompted us to investigate the gene here.

**TB1= TEOSINTE BRANCED 1; CYC= CYCLOIDEA PCF= PROLIFERATING* CELL FACTORS

Citations for Supplementary Table 1.

1. Rogers LA, Dubos C, Surman C, Willment J, Cullis IF, et al. (2005) Comparison of lignin deposition in three ectopic lignification mutants. New Phytol 168: 123–140. doi:10.1111/j.1469-8137.2005.01496.x.

2. Kandasamy MK, McKinney EC, Deal RB, Meagher RB (2005) Arabidopsis ARP7 is an essential actin-related protein required for normal embryogenesis, plant architecture, and floral organ abscission. Plant Physiol 138: 2019–2032. doi:10.1104/pp.105.065326.

3. Lee H, Suh SS, Park E, Cho E, Ahn JH, et al. (2000) The AGAMOUS-LIKE 20 MADS domain protein integrates floral inductive pathways in Arabidopsis. Genes Dev 14: 2366–2376.

4. Jofuku KD. BgwBMVMJKO (1994) Control of Arabidopsis Flower and Seed Development by the Homeotic Gene APETALA2. Plant Cell 6: 1211–1225. doi:10.1105/tpc.6.9.1211.

5. Mimida N, Goto K, Kobayashi Y, Araki T, Ahn JH, et al. (2001) Functional divergence of the TFL1-like gene family in Arabidopsis revealed by characterization of a novel homologue. Genes Cells 6: 327–336.

6. Reed JW (2001) Roles and activities of Aux/IAA proteins in Arabidopsis. Trends Plant Sci 6: 420–425.

7. Yang X, Lee S, So J-H, Dharmasiri S, Dharmasiri N, et al. (2004) The IAA1 protein is encoded by AXR5 and is a substrate of SCF(TIR1). Plant J 40: 772–782.

8. Stirnberg P (1999) AXR1 Acts after Lateral Bud Formation to Inhibit Lateral Bud Growth in Arabidopsis. Plant Physiol 121: 839–847. doi:10.1104/pp.121.3.839.

9. Yan H, Marquardt K, Indorf M, Jutt D, Kircher S, et al. (2011) Nuclear localization and interaction with COP1 are required for STO/BBX24 function during photomorphogenesis. Plant Physiol 156: 1772–1782. doi:10.1104/pp.111.180208.

10. Kumar R, Kushalappa K, Godt D, Pidkowich MS, Pastorelli S, et al. (2007) The Arabidopsis BEL1-LIKE HOMEODOMAIN proteins SAW1 and SAW2 act redundantly to regulate KNOX expression spatially in leaf margins. Plant Cell 19: 2719–2735. doi:10.1105/tpc.106.048769.

11. Schmitz G, Tillmann E, Carriero F, Fiore C, Cellini F, et al. (2002) The tomato Blind gene encodes a MYB transcription factor that controls the formation of lateral meristems. Proc Natl Acad Sci U S A 99: 1064–1069. doi:10.1073/pnas.022516199.

12. Aguilar-Martínez JA, Poza-Carrión C, Cubas P (2007) Arabidopsis BRANCHED1 acts as an integrator of branching signals within axillary buds. Plant Cell 19: 458–472. doi:10.1105/tpc.106.048934.

13. Lee J, Han C-T, Hur Y (2010) Overexpression of BrMORN, a novel “membrane occupation and recognition nexus” motif protein gene from Chinese cabbage, promotes vegetative growth and seed production in Arabidopsis. Mol Cells 29: 113–122. doi:10.1007/s10059-010-0006-2.

14. Fridman E, Carrari F, Liu Y-S, Fernie AR, Zamir D (2004) Zooming in on a quantitative trait for tomato yield using interspecific introgressions. Science 305: 1786–1789. doi:10.1126/science.1101666.

15. Dai Y, Wang H, Li B, Huang J, Liu X, et al. (2006) Increased Expression of MAP KINASE KINASE7 Causes Deficiency in Polar Auxin Transport and Leads to Plant Architectural Abnormality in ArabidopsisW. Plant Cell 18: 308–320.

16. Mulekar JJ, Bu Q, Chen F, Huq E (2012) Casein kinase II α subunits affect multiple developmental and stress-responsive pathways in Arabidopsis. Plant J 69: 343–354. doi:10.1111/j.1365-313X.2011.04794.x.

17. Manning K, Tör M, Poole M, Hong Y, Thompson AJ, et al. (2006) A naturally occurring epigenetic mutation in a gene encoding an SBP-box transcription factor inhibits tomato fruit ripening. Nat Genet 38: 948–952. doi:10.1038/ng1841.

18. Valverde F, Mouradov A, Soppe W, Ravenscroft D, Samach A, et al. (2004) Photoreceptor regulation of CONSTANS protein in photoperiodic flowering. Science 303: 1003–1006. doi:10.1126/science.1091761.

19. Almeida J, Rocheta M, Galego L (1997) Genetic control of flower shape in Antirrhinum majus. Development 124: 1387–1392.

20. Hirose N, Makita N, Kojima M, Kamada-Nobusada T, Sakakibara H (2007) Overexpression of a type-A response regulator alters rice morphology and cytokinin metabolism. Plant Cell Physiol 48: 523–539. doi:10.1093/pcp/pcm022.

21. Doi K, Izawa T, Fuse T, Yamanouchi U, Kubo T, et al. (2004) Ehd1, a B-type response regulator in rice, confers short-day promotion of flowering and controls FT-like gene expression independently of Hd1. Genes Dev 18: 926–936. doi:10.1101/gad.1189604.

22. Rajjou L, Belghazi M, Huguet R, Robin C, Moreau A, et al. (2006) Proteomic investigation of the effect of salicylic acid on Arabidopsis seed germination and establishment of early defense mechanisms. Plant Physiol 141: 910–923. doi:10.1104/pp.106.082057.

23. Chen L, Cheng JC, Castle L, Sung ZR (1997) EMF genes regulate Arabidopsis inflorescence development. Plant Cell 9: 2011–2024. doi:10.1105/tpc.9.11.2011.

24. Cong B, Barrero LS, Tanksley SD (2008) Regulatory change in YABBY-like transcription factor led to evolution of extreme fruit size during tomato domestication. Nat Genet 40: 800–804. doi:10.1038/ng.144.

25. Imaizumi T, Tran HG, Swartz TE, Briggs WR, Kay SA (2003) FKF1 is essential for photoperiodic-specific light signalling in Arabidopsis. Nature 426: 302–306. doi:10.1038/nature02090.

26. Hendry GAF (1993) Evolutionary origins and natural functions of fructans — a climatological , biogeographic and mechanistic appraisal. New Phytol 123: 3–14. doi:10.1111/j.1469-8137.1993.tb04525.x.

27. Nesbitt TC, Tanksley SD (2001) fw2.2 directly affects the size of developing tomato fruit, with secondary effects on fruit number and photosynthate distribution. Plant Physiol 127: 575–583.

28. Persson S, Caffall KH, Freshour G, Hilley MT, Bauer S, et al. (2007) The Arabidopsis irregular xylem8 mutant is deficient in glucuronoxylan and homogalacturonan, which are essential for secondary cell wall integrity. Plant Cell 19: 237–255. doi:10.1105/tpc.106.047720.

29. Huq E, Tepperman JM, Quail PH (2000) GIGANTEA is a nuclear protein involved in phytochrome signaling in Arabidopsis. Proc Natl Acad Sci U S A 97: 9789–9794. doi:10.1073/pnas.170283997.

30. Johnson CS, Kolevski B, Smyth DR (2002) TRANSPARENT TESTA GLABRA2, a Trichome and Seed Coat Development Gene of Arabidopsis, Encodes a WRKY Transcription Factor. Plant Cell 14: 1359–1375.

31. Caro E, Castellano MM, Gutierrez C (2007) GEM, a Novel Factor in the Coordination of Cell Division to Cell Fate Decisions in the Arabidopsis Epidermis. Plant Signal Behav 2: 494–495.

32. McCormac AC, Fischer A, Kumar AM, Söll D, Terry MJ (2001) Regulation of HEMA1 expression by phytochrome and a plastid signal during de-etiolation in Arabidopsis thaliana. Plant J 25: 549–561.

33. Takahashi Y, Shomura A, Sasaki T, Yano M (2001) Hd6, a rice quantitative trait locus involved in photoperiod sensitivity, encodes the alpha subunit of protein kinase CK2. Proc Natl Acad Sci U S A 98: 7922–7927. doi:10.1073/pnas.111136798.

34. Kobayashi Y, Kuroda K, Kimura K, Southron-Francis JL, Furuzawa A, et al. (2008) Amino acid polymorphisms in strictly conserved domains of a P-type ATPase HMA5 are involved in the mechanism of copper tolerance variation in Arabidopsis. Plant Physiol 148: 969–980. doi:10.1104/pp.108.119933.

35. Zhang Z-B, Yang G, Arana F, Chen Z, Li Y, et al. (2007) Arabidopsis inositol polyphosphate 6-/3-kinase (AtIpk2beta) is involved in axillary shoot branching via auxin signaling. Plant Physiol 144: 942–951. doi:10.1104/pp.106.092163.

36. Miyawaki K, Tarkowski P, Matsumoto-Kitano M, Kato T, Sato S, et al. (2006) Roles of Arabidopsis ATP/ADP isopentenyltransferases and tRNA isopentenyltransferases in cytokinin biosynthesis. Proc Natl Acad Sci U S A 103: 16598–16603.

37. Sureshkumar S, Todesco M, Schneeberger K, Harilal R, Balasubramanian S, et al. (2009) A genetic defect caused by a triplet repeat expansion in Arabidopsis thaliana. Science 323: 1060–1063. doi:10.1126/science.1164014.

38. Ormenese S, de Almeida Engler J, De Groodt R, De Veylder L, Inzé D, et al. (2004) Analysis of the spatial expression pattern of seven Kip related proteins (KRPs) in the shoot apex of Arabidopsis thaliana. Ann Bot 93: 575–580. doi:10.1093/aob/mch077.

39. Belles-Boix E, Hamant O, Witiak SM, Morin H, Traas J, et al. (2006) KNAT6: an Arabidopsis homeobox gene involved in meristem activity and organ separation. Plant Cell 18: 1900–1907. doi:10.1105/tpc.106.041988.

40. Greb T, Clarenz O, Schafer E, Muller D, Herrero R, et al. (2003) Molecular analysis of the LATERAL SUPPRESSOR gene in Arabidopsis reveals a conserved control mechanism for axillary meristem formation. Genes Dev 17: 1175–1187. doi:10.1101/gad.260703.

41. Svistoonoff S, Creff A, Reymond M, Sigoillot-Claude C, Ricaud L, et al. (2007) Root tip contact with low-phosphate media reprograms plant root architecture. Nat Genet 39: 792–796. doi:10.1038/ng2041.

42. Yaish MWF, Peng M, Rothstein SJ (2009) AtMBD9 modulates Arabidopsis development through the dual epigenetic pathways of DNA methylation and histone acetylation. Plant J 59: 123–135. doi:10.1111/j.1365-313X.2009.03860.x.

43. Demura T, Fukuda H (2007) Transcriptional regulation in wood formation. Trends Plant Sci 12: 64–70. doi:10.1016/j.tplants.2006.12.006.

44. Stirnberg P, van De Sande K, Leyser HMO (2002) MAX1 and MAX2 control shoot lateral branching in Arabidopsis. Development 129: 1131–1141.

45. Booker J, Auldridge M, Wills S, McCarty D, Klee H, et al. (2004) MAX3/CCD7 is a carotenoid cleavage dioxygenase required for the synthesis of a novel plant signaling molecule. Curr Biol 14: 1232–1238. doi:10.1016/j.cub.2004.06.061.

46. Sorefan K, Booker J, Haurogné K, Goussot M, Bainbridge K, et al. (2003) MAX4 and RMS1 are orthologous dioxygenase-like genes that regulate shoot branching in Arabidopsis and pea. Genes Dev 17: 1469–1474. doi:10.1101/gad.256603.

47. Mao C, Ding W, Wu Y, Yu J, He X, et al. (2007) Overexpression of a NAC-domain protein promotes shoot branching in rice. New Phytol 176: 288–298. doi:10.1111/j.1469-8137.2007.02177.x.

48. Huang J, Gu M, Lai Z, Fan B, Shi K, et al. (2010) Functional analysis of the Arabidopsis PAL gene family in plant growth, development, and response to environmental stress. Plant Physiol 153: 1526–1538. doi:10.1104/pp.110.157370.

49. Turner A, Beales J, Faure S, Dunford RP, Laurie DA (2005) The pseudo-response regulator Ppd-H1 provides adaptation to photoperiod in barley. Science 310: 1031–1034. doi:10.1126/science.1117619.

50. Noh Y-S, Amasino RM (2003) PIE1, an ISWI family gene, is required for FLC activation and floral repression in Arabidopsis. Plant Cell 15: 1671–1682.

51. Maloof JN, Borevitz JO, Dabi T, Lutes J, Nehring RB, et al. (2001) Natural variation in light sensitivity of Arabidopsis. Nat Genet 29: 441–446. doi:10.1038/ng777.

52. Casal JJ, Sánchez RA (1998) Phytochromes and seed germination. Seed Sci Res 8: 317–329.

53. Cerdán PD, Chory J (2003) Regulation of flowering time by light quality. Nature 423: 881–885. doi:10.1038/nature01636.

54. Filiault DL, Wessinger CA, Dinneny JR, Lutes J, Borevitz JO, et al. (2008) Amino acid polymorphisms in Arabidopsis phytochrome B cause differential responses to light. Proc Natl Acad Sci U S A 105: 3157–3162. doi:10.1073/pnas.0712174105.

55. Balasubramanian S, Sureshkumar S, Agrawal M, Michael TP, Wessinger C, et al. (2006) The PHYTOCHROME C photoreceptor gene mediates natural variation in flowering and growth responses of Arabidopsis thaliana. Nat Genet 38: 711–715. doi:10.1038/ng1818.

56. Halliday KJ, Whitelam GC (2003) Changes in photoperiod or temperature alter the functional relationships between phytochromes and reveal roles for phyD and phyE. Plant Physiol 131: 1913–1920. doi:10.1104/pp.102.018135.

57. Lazar G, Goodman HM (2006) MAX1, a regulator of the flavonoid pathway, controls vegetative axillary bud outgrowth in Arabidopsis. Proc Natl Acad Sci U S A 103: 472–476.

58. Peng J, Richards DE, Hartley NM, Murphy GP, Devos KM, et al. (1999) “Green revolution” genes encode mutant gibberellin response modulators. Nature 400: 256–261. doi:10.1038/22307.

59. Silverstone AL, Ciampaglio CN, Sun T (1998) The Arabidopsis RGA gene encodes a transcriptional regulator repressing the gibberellin signal transduction pathway. Plant Cell 10: 155–169.

60. Otsuga D, DeGuzman B, Prigge MJ, Drews GN, Clark SE (2001) REVOLUTA regulates meristem initiation at lateral positions. Plant J 25: 223–236.

61. Piskurewicz U, Jikumaru Y, Kinoshita N, Nambara E, Kamiya Y, et al. (2008) The gibberellic acid signaling repressor RGL2 inhibits Arabidopsis seed germination by stimulating abscisic acid synthesis and ABI5 activity. Plant Cell 20: 2729–2745.

62. Cazzonelli CI, Cuttriss AJ, Cossetto SB, Pye W, Crisp P, et al. (2009) Regulation of carotenoid composition and shoot branching in Arabidopsis by a chromatin modifying histone methyltransferase, SDG8. Plant Cell 21: 39–53. doi:10.1105/tpc.108.063131.

63. Konishi S, Izawa T, Lin SY, Ebana K, Fukuta Y, et al. (2006) An SNP caused loss of seed shattering during rice domestication. Science 312: 1392–1396. doi:10.1126/science.1126410.

64. Helariutta Y, Fukaki H, Wysocka-Diller J, Nakajima K, Jung J, et al. (2000) The SHORT-ROOT gene controls radial patterning of the Arabidopsis root through radial signaling. Cell 101: 555–567.

65. Welch D, Hassan H, Blilou I, Immink R, Heidstra R, et al. (2007) Arabidopsis JACKDAW and MAGPIE zinc finger proteins delimit asymmetric cell division and stabilize tissue boundaries by restricting SHORT-ROOT action. Genes Dev 21: 2196–2204.

66. Yamasaki K, Kigawa T, Inoue M, Tateno M, Yamasaki T, et al. (2004) A novel zinc-binding motif revealed by solution structures of DNA-binding domains of Arabidopsis SBP-family transcription factors. J Mol Biol 337: 49–63. doi:10.1016/j.jmb.2004.01.015.

67. Srivastava AC, Ganesan S, Ismail IO, Ayre BG (2008) Functional characterization of the Arabidopsis AtSUC2 Sucrose/H+ symporter by tissue-specific complementation reveals an essential role in phloem loading but not in long-distance transport. Plant Physiol 148: 200–211. doi:10.1104/pp.108.124776.

68. Tantikanjana T, Yong JW, Letham DS, Griffith M, Hussain M, et al. (2001) Control of axillary bud initiation and shoot architecture in Arabidopsis through the SUPERSHOOT gene. Genes Dev 15: 1577–1588. doi:10.1101/gad.887301.

69. Matser V, Davies B, Waites R (2010) The role of class I TCP genes in determining leaf shape and size. 21ST INTERNATIONAL CONFERENCE ON ARABIDOPSIS RESEARCH.

70. Sergeeva LI, Keurentjes JJB, Bentsink L, Vonk J, van der Plas LHW, et al. (2006) Vacuolar invertase regulates elongation of Arabidopsis thaliana roots as revealed by QTL and mutant analysis. Proc Natl Acad Sci U S A 103: 2994–2999. doi:10.1073/pnas.0511015103.

71. Kim D-H, Sung S (2010) Role of VIN3-LIKE 2 in facultative photoperiodic flowering response in Arabidopsis. Plant Signal Behav 5: 1672–1673.

72. NELSON OE, RINES HW (1962) The enzymatic deficiency in the waxy mutant of maize. Biochem Biophys Res Commun 9: 297–300.
